# Physiological demands of a swimming-based video game: Influence of gender, swimming background, and exergame experience

**DOI:** 10.1038/s41598-017-05583-8

**Published:** 2017-07-12

**Authors:** Pooya Soltani, Pedro Figueiredo, João Ribeiro, Ricardo J. Fernandes, João Paulo Vilas-Boas

**Affiliations:** 1 Centre of Research, Education, Innovation and Intervention in Sport (CIFI2D), Faculty of sport, University of Porto, Rua Dr Plácido Costa, 91, 4200-450 Porto, Portugal; 2 Department of Physical Education and Sport Sciences, School of Education and Psychology, Shiraz University, Pardis-e-Eram, Eram Square, 71946-84759 Shiraz Iran; 3 Porto Biomechanics Laboratory (LABIOMEP), University of Porto, Rua Dr Plácido Costa, 91, 4200-450 Porto, Portugal; 4 Department of Kinesiology, University of Maryland, College Park, MD USA

## Abstract

Active video games (exergames) may provide short-term increase in energy expenditure. We explored the effects of gender and prior experience on aerobic and anaerobic energy systems contributions, and the activity profiles of 40 participants playing with a swimming exergame. We recorded oxygen consumption and assessed blood lactate after each swimming technique. We also filmed participants’ gameplays, divided them into different phases and tagged them as active or inactive. Anaerobic pathway accounted for 8.9 ± 5.6% of total energy expenditure and although experienced players were less active compared to novice counterparts (η² < 0.15, p < 0.05), physiological measures were not different between performing groups. However, players with real-swimming experience during the first technique had higher heart rate (partial-η² = 0.09, p < 0.05). Our results suggest that short-term increase in physiological measures might happen in the beginning of gameplay because of unfamiliarity with the game mechanics. Despite low levels of activity compared to real sport, both aerobic and anaerobic energy systems should be considered in the evaluation of exergames. Game mechanics (involving the whole body) and strategies to minimize pragmatic play might be used for effective and meaningful game experience.

## Introduction

Higher screen times (e.g. playing videogames) are associated with physical inactivity^1^, and interventions to discourage their use are usually unsuccessful because players value these activities. Besides predicting parameters for increasing physical activity (PA) levels, playing sport videogames are associated with real sports participation among adolescents^2^. Newer generations of videogames (exergames) also provide opportunities for low to moderate (and sometimes large) energy expenditure (EE)^3^. Exergames are enjoyable and have group-play modes that make them potential tools in combatting common barriers to exercise. Mixed with traditional means of performing PA, exergames have also been shown to increase exercise satisfaction in obese children and offer alternatives for unmotivated participants to exercise regularly^4^, while having similar physiological effects^5^.

Depending on the videogame type and difficulty, exertion levels may vary, and higher PA intensities were observed when whole-body is involved during exergame play^6^. There are also mixed results regarding the effects of experience and gender on physiological parameters, with evidence suggesting that prior gaming experience does not affect mean heart rate (HR), but session rate of perceived exertion (RPE) and peak HR are higher among novice players^7^. Additionally, while it was shown that gaming experience may result in higher EE and oxygen uptake (${\dot{{\rm{V}}}{\rm{O}}}_{2}$)^8^, others mentioned that prior experience and resting HR do not affect EE during sport exergame play^6^. Similarly, gender was shown not to affect EE during exergaming among adults^9^, but others suggested that male players burn more energy^10^ and have higher ${\dot{{\rm{V}}}{\rm{O}}}_{2}$ and lower RPE compared to their female counterparts^11^. It should be noted that although playing time and number of playing bouts may not differ, boys play exergames more actively than girls^12^.

When playing exergames at moderate exercise intensity, and according to American College of Sports Medicine (ACSM) guidelines for health and fitness, aerobic energy pathway is believed to be the primary energy source. However, measuring only ${\dot{{\rm{V}}}{\rm{O}}}_{2}$ may neglect the role of glycolysis in total EE measurements^13^. Despite previous blood lactate (BLa) reports of 1.8 ± 0.8 mmol.l^−1^ for an upper-body exergame (boxing) and 2.4 ± 1.5 mmol.l^−1^ for a lower-body computer game^14^, BLa was never considered in the assessment of EE. This consideration is important as sports exergames are meant to replicate real sports and their physiological demands, which during its design phase might ensure a more meaningful experience.

Although HR, RPE, movement monitoring and ${\dot{{\rm{V}}}{\rm{O}}}_{2}$ are among popular intensity measurements^15^, newer methodologies tried to estimate metabolic energy cost using algorithmic models^16^. As many exergame platforms provide feedback on EE estimation based on specific formulas, considering the anaerobic energy pathway might also be useful in improving their accuracy. Researchers may also use time-motion analysis as an indirect method for estimating physiological stress, particularly by dividing the game into sub-activities. This objective assessment of exergames could be used when normal physiological measurements are intrusive^17^. As performing a short effort activity requires using a different metabolic pathway compared to longer activities^18^, time-motion analysis may also provide information on including the right energy system.

Swimming is a well-practiced and appreciated PA, and a simulating swimming game might be an alternative for those who do not have access to a swimming pool. Competing against the virtual multi-medallist Michael Phelps, might be a motivating and challenging once in a lifetime experience. Since no research was conducted to measure the relative contribution of the anaerobic energy system to total EE in exergame playing, the purpose of this study was to characterise the total energy demands (aerobic and anaerobic) and activity profiles in a swimming exergame. In addition, we compared the physiological demands of groups with different experience and gender. We hypothesised that experienced players, non-real swimmers and female players would have lower physiological characteristics and lower activity time during the gameplay.

## Results

**Figure 1:**
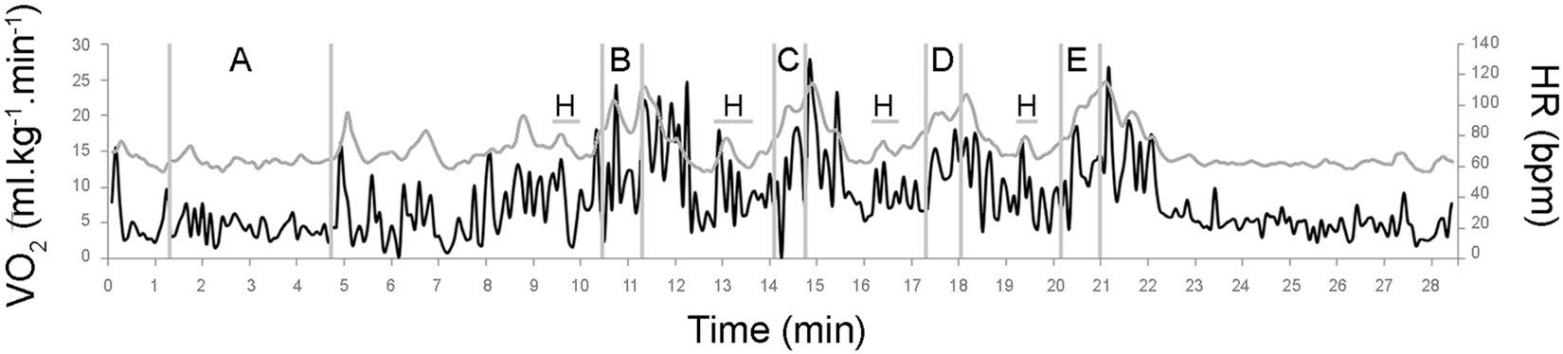
Typical oxygen consumption (black line) and heart rate (grey line) behaviour during a bout of swimming exergame during the rest period (**A**), front crawl (**B**), backstroke (**C**), breaststroke (**D**), butterfly (**E**) and Hype phase (**H**).

For all subjects, we observed ${\dot{{\rm{V}}}{\rm{O}}}_{2{\rm{rest}}}$ ${\dot{{\rm{V}}}{\rm{O}}}_{2{\rm{rest}}}$ of 4.9 ± 1.1 and ${\dot{{\rm{V}}}{\rm{O}}}_{2{\rm{peak}}}$ of 25.7 ± 6.0 ml.kg^−1^.min^−1^, [La^−^]_rest_ of 1.4 ± 0.6 mmol.l^−1^, HR_rest_ of 67.9 ± 17.0 beats per minute (bpm), and ${\dot{{\rm{V}}}{\rm{O}}}_{2{\rm{peak}}}$ of 21.4 ± 6.4 during the front crawl, 20.72 ± 5.43 during backstroke, 18.21 ± 5.33 during breaststroke and 18.66 ± 6.44 ml.kg^−1^.min^−1^ during butterfly. Figure 1 presents an example of ${\dot{{\rm{V}}}{\rm{O}}}_{2}$ kinetics during a typical game session for a subject. The values of physiological measurements are reported in Table 1.

**Table 1:** Mean ± SD physiological parameters and activity profile in different exergame performing groups.

Variables		Swimming experience		Exergame experience		Gender	
		Swimmer (N=16)	Non-swimmer (N=24)	Experienced (N=24)	Novice (N=16)	Male (N=31)	Female (N=9)
Age		24.1 ± 4.9	23.7 ± 4.2	24.3 ± 5.1	23.1 ± 3.2	23.3 ± 3.7	25.5 ± 6.3
Height (cm)		173.9 ± 8.3	174.0 ± 6.3	173.5 ± 7.2	174.7 ± 7.0	176.2 ± 5.8	166.1 ± 5.1
Body mass (kg)		71.6 ± 11.4	72.0 ± 11.2	71.5 ± 9.7	72.4 ± 13.4	74.6 ± 1.7	62.4 ± 6.8
BLa (mmol.l^−1^)	BLa_ACTIVITY_	3.0 ± 1.4	2.3 ± 0.8	2.4 ± 1.0	3.0 ± 1.3	2.7 ± 1.2	2.1 ± 0.5
	Crawl	3.0 ± 3.0	2.0 ± 0.7	2.1 ± 1.6	3.0 ± 2.7	2.5 ± 2.3	2.0 ± 0.7
	Backstroke	2.7 ± 1.0	2.1 ± 0.8	2.2 ± 0.9	2.7 ± 1.0	2.5 ± 1.0	1.9 ± 0.6
	Breaststroke	3.0 ± 0.9	2.5 ± 0.6	2.6 ± 0.7	3.0 ± 0.9	2.8 ± 0.8	2.3 ± 0.5
	Butterfly	3.3 ± 2.1	2.7 ± 1.4	2.6 ± 1.4	3.5 ± 2.1	3.1 ± 1.9	2.4 ± 0.8
EE (kJ)	EE_TOTAL_	113.4 ± 40.4	97.4 ± 24.1	95.3 ± 24.4	119.3 ± 39.5	111.0 ± 33.5	82.2 ± 15.7
	EE_LAC_	12.9 ± 11.6	7.8 ± 5.0	7.9 ± 6.7	13.5 ± 10.6	11.3 ± 9.4	5.7 ± 2.7
	EE_AER_	100.5 ± 32.8	89.6 ± 23.0	87.4 ± 22.4	105.8 ± 32.6	99.7 ± 28.6	76.4 ± 14.6
	Lactic (%)	10.2 ± 6.6	8.0 ± 4.8	8.0 ± 5.5	10.5 ± 5.8	9.6 ± 6.2	6.8 ± 2.7
	Aerobic (%)	89.7 ± 6.6	91.9 ± 4.8	91.9 ± 5.5	89.4 ± 5.8	90.3 ± 6.2	93.0 ± 2.7
HR (bpm)	HR_TOTAL_	94.1 ± 18.3	85.5 ± 12.5	88.4 ± 16.9	89.8 ± 13.5	88.3 ± 15.6	91.2 ± 15.4
	HR_ACTIVITY_	105.7 ± 15.7	97.9 ± 13.9	99.0 ± 13.1	104.0 ± 17.5	99.2 ± 14.7	107.3 ± 15.1
	Crawl	105.9 ± 17.9*	96.8 ± 11.8	100.0 ± 15.3	101.1 ± 15.1	98.8 ± 15.8	106.1 ± 11.0
	Backstroke	105.8 ± 13.8	103.0 ± 16.6	101.6 ± 12.0	108.0 ± 19.4	102.1 ± 14.9	111.1 ± 16.3
	Breaststroke	105.4 ± 17.8	99.3 ± 16.9	96.9 ± 15.0	104.5 ± 19.6	98.2 ± 17.0	106.0 ± 17.3
	Butterfly	106.0 ± 15.8	98.2 ± 16.9	97.8 ± 15.5	103.7 ± 17.0	98.3 ± 16.3	106.4 ± 18.8
RPE	RPE_ACTIVITY_	2.9 ± 1.1	3.0 ± 1.2	2.8 ± 1.2	3.2 ± 1.2	2.9 ± 1.2	3.2 ± 1.4
	Crawl	2.6 ± 1.3	2.0 ± 1.2	2.1 ± 1.3	2.4 ± 1.2	2.2 ± 1.3	2.2 ± 1.3
	Backstroke	2.8 ± 1.0	3.0 ± 1.6	2.6 ± 1.3	3.4 ± 1.5	2.7 ± 1.3	3.6 ± 1.5
	Breaststroke	3.0 ± 1.4	3.2 ± 1.5	3.0 ± 1.5	3.3 ± 1.5	3.0 ± 1.4	3.5 ± 1.6
	Butterfly	3.4 ± 1.7	4.0 ± 1.5	3.6 ± 1.7	3.9 ± 1.3	3.8 ± 1.5	3.6 ± 1.9
Activity profile	Active (%)	54.5 ± 4.4	58.5 ± 9.5	55.8 ± 8.6	58.6 ± 7.1	56.4 ± 7.2	58.5 ± 10.9
	Rest (%)	44.3 ± 5.0	44.0 ± 7.8	45.5 ± 7.2	42.0 ± 5.4	43.3 ± 6.1	47.0 ± 8.3
	E:R	1.2 ± 0.2	1.3 ± 0.4	1.2 ± 0.3	1.4 ± 0.3	1.3 ± 0.3	1.2 ± 0.2

Aerobic: relative aerobic percentage; BLa: blood lactate; EE: energy expenditure; EE_AER_: aerobic energy contribution; EE_LAC_: anaerobic energy contribution; EE_TOTAL_: total energy expenditure; E:R: effort to rest ratio; HR: heart rate; HR_ACTIVITY_: mean HR during the four swimming events; Lactic: relative anaerobic lactic percentage; HR_TOTAL_: HR from the onset of activity until the end of the last technique; N: number; RPE: rate of perceived exertion; RPE_ACTIVITY_: mean RPE during the four swimming technique.

Mean BLa during the activity was 2.6 ± 1.1 mmol.l^−1^ and was not different between performing groups (p > 0.05, partial-η² < 0.05). Peak BLa was 2.9 ± 1.3 mmol.l^−1^ and occurred 3 min after the end of the gameplay. Mean EE during the activity was 104.2 ± 32.5 kJ (94.2 ± 27.6 kJ aerobic plus 9.9 ± 8.6 kJ anaerobic) and was not different between performing groups (p > 0.05, partial-η² < 0.09). The lactic pathway accounted for 8.9 ± 5.6% of total EE. Figure 1 illustrates a typical HR change throughout the gameplay. Mean HR during the gameplay was 101.0 ± 14.8 bpm, corresponding to 49.9 ± 21.6% above the resting HR and 51.5 ± 7.4% of maximum HR. Only participants with real-swimming experience had higher values compared to non-swimmers during front crawl event (F(1, 38) = 3.78, partial-η² = 0.09, p = 0.04). Mean RPE during the activity was 3.0 ± 1.2 and was not different between performing groups (p > 0.05, partial-η² < 0.01). There were also no interactions between swimming experience, game experience and gender on BLa, EE, HR and RPE changes (p > 0.05).

**Figure 2:**
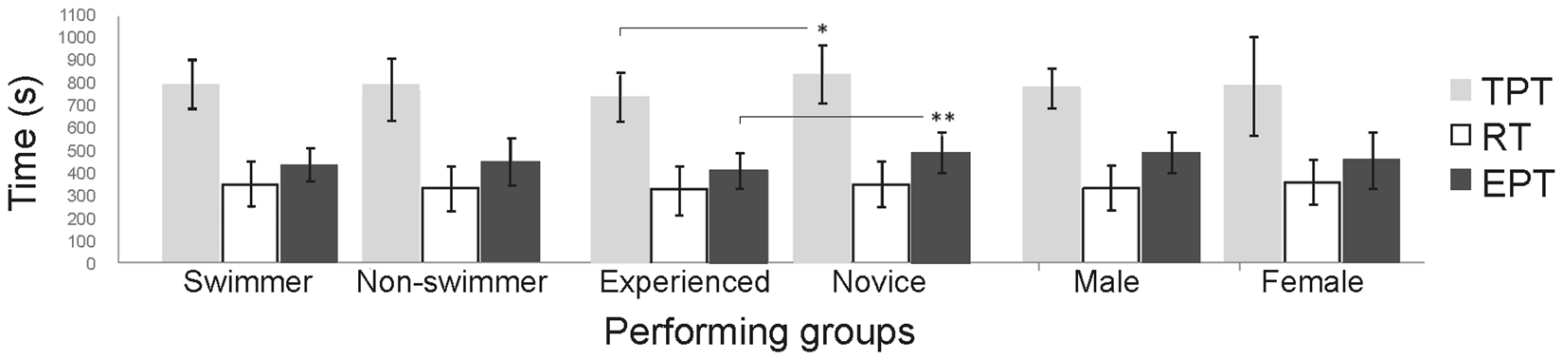
Mean TPT, RT and EPT in different performing groups (TPT: total playing time; RT: rest time; EPT: effective playing time; * and **: differences between TPT and EPT).

While we measured a high intra-observer reliability of 0.96 for time-motion analysis, a second reliability check was also performed implementing TEM (with 95% confidence interval - CI) for each variable as follows: mean activity time of 441 s (95% CI = 421–450 s) and rest time of 287 s (95% CI = 267–296 s). The relative TEM of 3.5% was within the acceptable range^19^. Players were active 56.9 ± 8.1% (range 42.7–85.1%) of the total time, rested 44.2 ± 6.8% (range 27.7–64.7%) and had E:R of 1.3 ± 0.3 during the gameplay. No differences were found between the performing groups (η² > 0.01, p > 0.05). Figure 2 highlights the mean duration of TPT, RT and EPT within different performing groups. Previous exergame experience (experienced vs. novice) resulted in lower TPT (743 vs. 844 s; F(1, 38) = 6.86, partial-η² = 0.15, p = 0.01), EPT (413 vs. 495 s; F(1, 38) = 8.70, η² = 0.18, p = 0.01) and E:R (mean rank 17.7 vs. 24.6; χ^2^(1) = 3.422, p = 0.05). No interaction was observed between performing groups and TPT, RT and EPT (p > 0.05).

## Discussion

The aims of this study were to estimate different energy systems’ contributions, to provide an activity profile of gameplay and to compare the results in different performing groups. Anaerobic pathway accounted for 8.9% of total energy production and players were active 57% of total gameplay. Performing groups did not have different BLa and RPE, and mean HR was only higher in participants with real-swimming experience during the crawl event. Experienced players also had lower TPT, EPT and E:R ratio compared to the novice players.

Our obtained ${\dot{{\rm{V}}}{\rm{O}}}_{2{\rm{peak}}}$ values were similar to the previous reports^3^. However, these values may be affected by game mechanics, game duration and participants’ performing levels. Higher ${\dot{{\rm{V}}}{\rm{O}}}_{2{\rm{peak}}}$ values during front crawl might have occurred as it was the first technique and participants were trying to swim close to real-swimming technique. Moreover, ${\dot{{\rm{V}}}{\rm{O}}}_{2{\rm{peak}}}$ during front crawl was also lower than real-swimming with full body and upper body^20^. Alternatively, lower ${\dot{{\rm{V}}}{\rm{O}}}_{2}$ during breaststroke could be explained by the lower range of motion (activity) of the upper-limbs. Additionally, as there were almost no forces applied on the body compared to in-water hydrostatic pressure, different responses were expectable.

Mean BLa in our study was higher than findings of Jordan *et al*.^14^ probably due to different game design, recruited muscles, type of platform, intensity and the duration of the gameplay. BLa values of performing groups were similar and low percentage of their variabilities, accounted by the different performing categories. While, we reject our hypothesis stating that real swimmers, experienced and female players had lower BLa during the gameplay, we should state that this might have happened as the gaming platform detect different movement patterns of players similarly, and players may switch to pragmatic gameplay even after a short exposure to the game.

As participants had to use both upper-limbs during the gameplay, EE levels were higher than sports exergames using only one upper-limb (e.g. tennis, bowling)^21^ but lower than games incorporating both upper and lower limbs^14^ and real-swimming^20^. Possible explanations lie within the different design (incorporating different muscle groups), different EE measurement methodologies, different demands of gaming platform and efficient interaction with the gaming platform. EE was also similar between groups and the low percentage of its variability accounted by different performing groups. Contrary to the previous research, higher EE in novice players might have also occurred because of longer gameplay, as they spent more time to complete the events. Moreover, players with real-swimming experience might have put more effort swimming correctly (according to the real-world techniques) at the beginning of their gameplay or during the first technique. Contrary to the previous research^10^, in our study male and female players did not have different EE and HR.

We also obtained higher values of HR compared to the previous study on Wii muscle conditioning and brisk walking^22^. We also reject our hypothesis stating that real swimmers, experienced and female players had lower HR compared to their counterparts. Additionally, RPE was not different within any performing groups, and the values were also lower compared to previous research in full-body and upper-body exergames^23^. Our results also suggest that the type of gaming platform (Xbox, Wii, etc.) does not lower psychological perception of exertion^5^, and although novice players played the game for a longer time, RPE was not different from experienced players. This was consistent with previous research suggesting that immersive exergames may alter players’ perception of game intensity resulting in longer gameplay^24^. Therefore, we reject our hypothesis stating that real swimmers, experienced and female players had lower RPE than their peers.

The average effort to rest ratio in the current study was 1.3 ± 0.3, showing that although players dedicated more time playing than resting, the results were not statistically different. Our active play values were also lower than previous study, ranging from 65–88%^3^. Possible reasons are lengthy waiting times between each bout and low activity times during each technique. While novice and experienced players did not differ in RT, experienced players spent less time playing with the game. Shorter playing time might have happened due to navigating faster through the menus and following game strategies in experienced players. Therefore, we reject our hypothesis that experienced players, real swimmers and female players had higher RT compared to their peers.

As it may not be possible to reduce video game playing completely, proper exergame design might still increase PA levels. Identification of work and rest intervals could provide relevant data on how to encourage players to expend more energy in a more realistic manner. As fast game play might be used as a strategy to encourage players to be more active and stimulate excitement, measuring anaerobic pathway behaviour might be used in balancing the activities to avoid boredom and hasty fatigue. Moreover, if the obtained effort to rest ratio is compared with other games, it can potentially be used as a fitness index for exergames. The results of this study are useful for user experience researchers, game designers, and physical educators who want to apply exergame in their practice. Scientific and descriptive information of movement patterns and physiological characterization of exergames are necessary for designing effective fitness experience and game design. Software loading and menu selection have great effects on increasing workout times^3^, and by using auditory commands, using bigger icons and default presets, such timings could be shortened leading to an increase in effective gameplay. Future studies might use larger sample size for each performing group (e.g. gender) to ensure statistically significant differences of physiological variables between performing groups.

## Conclusions

We have quantified several physical and technical variables to explore the physical demands of exergame playing in more detail, which provide foundations for developing specific exergames. We showed that short-term increase in physiological measures might have happened because of unfamiliarity with exergames and as players understand the game mechanics, they might exert less while playing. Despite low levels of activity compared to real sport, both energy systems should be considered in EE measurements of exergames. Various performing groups did not respond to the game differently, because players’ movements were detected similarly by the gaming platform. Moreover, the current investigation suggests using time-motion analysis during game design to increase the exercise to rest ratio.

## Methods

Forty participants (9 females, age 23.8 ± 4.4 years, height 174.0 ± 7.1 cm, body mass 71.9 ± 11.2 kg) participated in the study, which was approved by the local ethics committee (CEFADE 01/2013) and performed according to the Declaration of Helsinki. Participants signed informed consents and were asked to avoid strenuous activity and smoking 24 h before the testing, to drink water liberally and to refrain from consuming alcohol, caffeine and food, at least 2 h before their participation. We considered participants who had played this game before as experienced (6 females) and those who knew, at least, two conventional swimming techniques were considered as swimmers (4 females).

The exercise task was a swimming exergame designed for Microsoft Xbox360 and Kinect, offering four swimming techniques (Michael Phelps: Push the Limit, 505 Games, Italy). Each participant had to stand in front of the Kinect sensor and move their upper-body according to front crawl, backstroke, breaststroke and butterfly swimming techniques to move the avatar inside the game, competing against the computer opponent. No instruction was provided on how to play the game. However, as part of the game and before participation, each player watched an in-game trial video on how to play the game. There was no familiarisation with the game itself, but players were given the chance to navigate between the menus of the game and explore the features of the game. Each 100 m event was controlled by an on-screen visual feedback, preventing players from swimming too fast or too slow, and in the middle of the second 50 m lap, there was a possibility of swimming as fast as possible (Push the Limit – PTL). Oxygen uptake at rest (${\dot{{\rm{V}}}{\rm{O}}}_{2{\rm{rest}}}$) and heart rate at rest (HR_rest_) values were obtained, and to avoid varying work rate increments, the order of events was equal for all participants. Breath-by-breath ${\dot{{\rm{V}}}{\rm{O}}}_{2}$ was measured using a portable analyser (K4b^2^, Cosmed, Italy) and BLa (25 µl) were obtained from the earlobe (Lactate Pro, Arkay Inc, Japan) at rest (BLa_rest_), immediately after completion of each swimming technique and 3, 5 and 7 min following the gameplay or until the maximum value was obtained. The difference in lactate accumulation after and before activity (BLa_net_) was measured as the differences between BLa at the end of the last event and BLa_rest_, allowing estimating the partial contribution of anaerobic energy pathway. RPE was administered using OMNI (0–10) immediately after each technique^25^.

We verified ${\dot{{\rm{V}}}{\rm{O}}}_{2}$ data and deleted irregular values from the analysis (considering only values in-between mean ± 4SD). We smoothed the ${\dot{{\rm{V}}}{\rm{O}}}_{2}$ recordings using a 3-breath moving average and time-averaged at 5 s intervals^13^. Following that, we recorded peak oxygen uptake (${\dot{{\rm{V}}}{\rm{O}}}_{2{\rm{peak}}}$) during the exercise and calculated aerobic energy contribution from the time integral of net ${\dot{{\rm{V}}}{\rm{O}}}_{2}$ versus time relationship^13^. We calculated the anaerobic lactic contribution (AnL) using Equation 1.

1 $${\rm{AnL}}=\beta \times {{\rm{BLa}}}_{{\rm{net}}}\times M$$

where: BLa_net_ is the difference in lactate accumulation after and before activity, *β* is the energy equivalent of BLa accumulation (2.7 ml.O_2_.mM^−1^.kg^−1^)^26^, and *M* is the mass of the participant. To express the EE in kJ during aerobic and anaerobic lactic energy contributions, an energy equivalent of 20.9 kJ.l.O_2_^−1^ was assumed^27^.

We also filmed players’ gameplays, divided the video recordings and tagged them as active and rest (inactive) to an accuracy of 1 s, based on Table 2, using a video edit software (Movie Edit Pro, Magix AG, Germany). We marked the beginning and end of each movement, and the duration of each action was measured, to calculate total playing time (TPT), effective playing time (EPT), resting time (RT) and effort to rest ratio (E:R).

**Table 2:** Coding front crawl technique during swimming exergame for notational analysis.

Phase	Tag	Description
Introduction	RT	Stepping in front of the sensor.
Shake to play	EPT	Shaking hand in a semi-circular movement for the sensor to detect the player.
Profile selection	EPT	Player signs in with his/her gaming preferences or as a guest.
Position adjustment	RT	Visual feedback to ask the player to stand within the visibility of the Kinect.
Mode and technique	EPT	Selecting single player or multi player and type of swimming technique.
Press start	EPT	Pressing start button.
Presentation	RT, Skip	Player watches a brief video instruction about how to play the game.
Loading	RT	Player waits for the game to be loaded.
Hype the crowd	RT, EPT, Skip	Players can move their body to cheer the audience and get extra points. In fact,
Start	EPT	Player bends forward with both hands in front. After seeing the audio-visual command, they extend their back and stand with their hands in front (In backstroke, the start includes bending the knees with holding hands in front, extending the knees, standing and raising the hands up after the audio-visual command).
Swim	EPT	Swimming according to the technique.
Return	RT, EPT	Extending one arm after seeing the visual feedback to reach to an imaginary wall and return.
Swim	EPT	Continuing the swimming according to the technique.
Push the limit (PTL)	EPT	Player could swim without any feedback and as fast as possible.
End	EPT	Player drops both arms and raises one arm to terminate the race.
Continue to game	EPT	Player selects another technique.

EPT: effective playing time; RT: resting time; Skip: the player has the options to rest, play, or skip this part using auditory commands.

We reported descriptive statistics for all variables and checked the normality using Shapiro-Wilk. We used a one-way analysis of variance (ANOVA) to compare physiological and temporal parameters during each event and within performing groups. In the case of violation of homogeneity of variance, we utilised alternative non-parametric statistics of Kruskal-Wallis H. We also used a three-way ANOVA to determine the effect between three performing groups and their interaction effect on BLa, EE, HR and RPE. We utilised SPSS 23 (Chicago, IL) and set the significance level to p < 0.05. To assess the practical significance of the findings, we computed an effect size for each analysis using the eta-squared statistics (η²). We also established the reproducibility of the time-motion analysis using Lin’s Concordance Coefficient^28^. Two participants were randomly chosen and analysed twice by the same researcher, and the technical error of measurement (TEM) for intra-evaluator test-retest was measured for the performance variables (rest and activity)^29^. To avoid retention of knowledge of the content, the retest analysis was conducted one month after the initial testing. TEM accuracy estimations are shown as 95% confidence limits using Equation 2 ^30^.

2 $${\rm{Absolute}}\,{\rm{TEM}}=\sqrt{\frac{{\sum }^{}{{\rm{d}}}^{2}}{2{\rm{n}}}}$$

where: d is the deviations between the two measurements and n is the number of deviations. We then transformed the absolute TEM into relative TEM, to express the error in percentages, using Equation 3 ^31^, where: VAV is the variable average value (expressed as the sum of the two measurements divided by two).

3 $${\rm{Relative}}\,{\rm{TEM}}=\frac{{\rm{TEM}}}{{\rm{VAV}}}\times 100$$

### Practical implications

- Experienced exergame players are less active than novice players.
- Both aerobic and aerobic energy systems should be used in energy expenditure measurement of exergaming.
- Short-term increase in physiological measures in exergames might happen because of unfamiliarity of players.

## References

[1] Christofaro DGD (2016). Higher screen time is associated with overweight, poor dietary habits and physical inactivity in Brazilian adolescents, mainly among girls. Eur J Sport Sci..

[2] Adachi PJC, Willoughby T (2016). Does playing sports video games predict increased involvement in real-life sports over several years among older adolescents and emerging adults?. J Youth Adolescence..

[3] Bronner S, Pinsker R, Noah JA (2013). Energy cost and game flow of 5 exer-games in trained players. Am J Health Behav..

[4] Finco MD (2015). Exergaming as an alternative for students unmotivated to participate in regular physical education classes. International Journal of Game-Based Learning..

[5] Lisón JF (2015). Competitive active video games: Physiological and psychological responses in children and adolescents. Paediatr Child Healt..

[6] Wu PT, Wu WL, Chu IH (2015). Energy Expenditure and Intensity in Healthy Young Adults during Exergaming. Am J Health Behav..

[7] Kraft JA (2015). Influence of experience level on physical activity during interactive video gaming. J Phys Act Health..

[8] Bonetti AJ (2010). Comparison of acute exercise responses between conventional video gaming and isometric resistance exergaming. J Strength Cond Res..

[9] Miyachi M (2010). METs in adults while playing active video games: a metabolic chamber study. Med Sci Sports Exerc..

[10] Sit CH, Lam JW, McKenzie TL (2010). Direct observation of children’s preferences and activity levels during interactive and online electronic games. J Phys Act Health..

[11] Graf DL (2009). Playing active video games increases energy expenditure in children. Pediatrics..

[12] Lam JWK, Sit CHP, McManus AM (2011). Play pattern of seated video game and active “exergame” alternatives. J Exerc Sci Fit..

[13] Sousa, A. C., Vilas-Boas, J. P. & Fernandes, R. J. Kinetics and metabolic contributions whilst swimming at 95, 100, and 105% of the velocity at VO_2max_. *BioMed Res. Int*. **2014**, Article ID 675363, 9 pages doi:10.1155/2014/675363 (2014). 10.1155/2014/675363 PMC4087294 25045690

[14] Jordan M, Donne B, Fletcher D (2011). Only lower limb controlled interactive computer gaming enables an effective increase in energy expenditure. Eur J Appl Physiol..

[15] Gao Z (2015). A meta-analysis of active video games on health outcomes among children and adolescents. Obes Rev.

[16] Nathan D (2015). Estimating physical activity energy expenditure with the Kinect sensor in an exergaming environment. PloS one..

[17] Hughes, M. & Franks, I. M. (Eds.). *Notational analysis of sport: Systems for better coaching and performance in sport*. (Psychology Press, 2004).

[18] Gastin PB (2001). Energy system interaction and relative contribution during maximal exercise. Sports Med..

[19] Duthie G, Pyne D, Hooper S (2003). The reliability of video based time motion analysis. J. Hum. Mov. Stud..

[20] Ribeiro J (2015). VO_2_ kinetics and metabolic contributions during full and upper body extreme swimming intensity. Eur J Appl Physiol..

[21] Graves LEF, Ridgers ND, Stratton G (2008). The contribution of upper limb and total body movement to adolescents’ energy expenditure whilst playing Nintendo Wii. Eur J Appl Physiol..

[22] Graves LEF (2010). The physiological cost and enjoyment of Wii fit in adolescents, young adults, and older adults. J Phys Act Health..

[23] Whitehead, A. *et al*. *Exergame effectiveness: what the numbers can tell us*. Paper presented at the Proceedings of the 5th ACM SIGGRAPH Symposium on Video Games. (Los Angeles, California, 2010).

[24] Lau P (2015). Evaluating physical and perceptual responses to exergames in chinese children. Int J Env Res Pub He..

[25] Irving BA (2006). Comparison of Borg-and OMNI-RPE as markers of the blood lactate response to exercise. Med Sci Sports Exerc..

[26] di Prampero, P. *et al*. Blood lactic acid concentrations in high velocity swimming in *Swimming**Medicine IV* (Eds. B. Eriksson, B. & Furberg, B.) 249–261 (University Park Press, 1978).

[27] Figueiredo P (2011). An energy balance of the 200 m front crawl race. Eur J Appl Physiol..

[28] Lawrence I, Lin K (1989). A concordance correlation coefficient to evaluate reproducibility. Biometrics..

[29] Hopkins WG (2000). Measures of reliability in sports medicine and science. Sports Med..

[30] Tanner, R. & Gore, C. *Physiological tests for elite athletes* (Human Kinetics, 2013).

[31] Perini TA (2005). Technical error of measurement in anthropometry. Revista Brasileira de Medicina do Esporte..

